# 16-year follow-up of Family Spirit intervention effects on Native American maternal-child outcomes: study protocol

**DOI:** 10.1186/s12889-026-28261-9

**Published:** 2026-07-17

**Authors:** Katie E. Nelson, Emily Haroz, Nicole Neault, Novalene Goklish, Valerie Coho-Mescal, Sherilynn Lorenzo, Jennifer Richards, Tamara Talaswaima, Timothy R. Werwie, Meenakshi Richardson, Vergilene Velasquez-Brawley, Allison Barlow

**Affiliations:** Department of International Health, Johns Hopkins Bloomberg School of Public Health, 415 N Washington ST. 4th Floor, Baltimore, MD 21231 USA

**Keywords:** American Indian, Parenting Interventions, Home-visiting, Substance use, Mental Health, Long-term-follow-up

## Abstract

**Background:**

*Family Spirit* is a culturally grounded home-visiting program, delivered by paraprofessional home visitors, which was designed for and has been proven effective with Native American families. A randomized controlled trial of the Family Spirit intervention was conducted between 2006 and 2011, yielding positive outcomes for young mothers and their children at 3 years post-partum. This 16-year long-term follow-up study, to be conducted between 2023 and 2028, aims to determine if Family Spirit’s early impacts resulted in long-term protection against problematic substance use and suicide during peak risk periods. Both substance use and suicide are leading causes of death and despair for Native Americans, resulting in significant public health disparities for Native American communities.

**Methods:**

This study will take place in the four communities that participated in the original Family Spirit trial. The study aims to recruit at least 75% (*n* = 242) of the mothers and their now adult children (*n* = 242) who participated in the original trial (*n* = 322). We will use an explanatory mixed-methods design, involving self-report questionnaires and electronic health record reviews to understand Family Spirit’s long-term impacts on maternal and child substance use, suicide, and related consequences, as well as mediators and moderators of these outcomes. Additionally, semi-structured in-depth interviews with a purposively sampled group of participants will be used to understand the intervention’s long-term impacts on individual and intergenerational experiences surrounding substance use, suicide, and related consequences.

**Discussion:**

This study will address critical knowledge gaps in understanding the long-term effectiveness of early childhood home-visiting programs, especially among Native American communities. This is the first long-term follow-up study of a home visiting intervention to assess substance use and suicide impacts directly, and the first to document long-term efficacy of paraprofessional home visitors. In summary, this study presents a unique and important opportunity to assess whether a culturally grounded early childhood home-visiting intervention provides long-term protection during a timeframe of peak mortality risk.

**Trial registration:**

ClinicalTrials.gov, TRN: NCT00373750; Registration date: 12 January 2015.

## Background

Native American (NA) (American Indian/Alaska Native) communities have long maintained strong traditions of kinship and cultural practices that guide positive child-rearing and promote long-term holistic health [1, 2]. Traditional knowledge systems and communal practices are inherently protective for intergenerational well-being, creating resilient communities with sophisticated approaches to child development and health rooted in cultural identity and connection [3, 4]. However, these protective ways have been systematically disrupted through colonization and federal policy that has contributed to pervasive historical and contemporary traumas [5].

The organized destruction of NA lifeways has resulted in profound health disparities [6]. Historical trauma within NA communities has compromised parents’ and families’ capacity to access and utilize traditional parenting practices. The earliest effects of disrupted parenting result in infant and toddler externalizing and internalizing problems [7, 8], which are risk factors for drug use [9]; anti-social tendencies [7], delinquency and aggressive behavior [9]; high-risk sex and unplanned pregnancy [10]; anxiety, depression, and suicide in adolescence [8, 11, 12]. Maternal drug use, depression, and multi-generational adversity also contribute to their children’s drug use [13].

Recent national mortality data highlight urgent public health disparities. NA communities now experience the most dramatic increases in premature mortality of any racial/ethnic group, with annual mortality increases of 4.3% per year among NA women aged 30—the highest rate of any demographic group between 1999 and 2014 [14]. NA women of childbearing age currently have the highest overdose rates of any female racial group in the U.S.—twice the rate of white women ages 20-39 [15]. NA youth suicide rates peak at age 20 and again between 30 and 40 years, and projections indicate these trends will continue, with NA populations expected to have the highest accidental mortality rates by 2030: 97.5 deaths per 100,000 women and 298.7 deaths per 100,000 men [16].

Early childhood home-visiting is a promising strategy for leveraging protective family practices early in life with the goal of sustaining long-term wellness. The Family Spirit Program is the only one of 24 federally endorsed home-visiting models designed for and proven effective with NA communities [17–19]. Beginning in 1995, Family Spirit was created through community partnerships that honor Indigenous knowledge systems, while addressing contemporary challenges and building upon community assets. Participating communities from three partnering Tribal Nations engaged in every stage of Family Spirit curriculum development. A cross-site Community Advisory Board (CAB) advised that home-visiting should target teen-formed families to address intergenerational poverty cycles and overcome barriers to clinic-based care. While evidence supported nurse-delivered models, the CAB selected paraprofessional home visitors from local communities to build rapport with multi-generational families requiring bilingual communication. Direct community input guided curriculum content development and possible evaluation study designs [18, 20]. Ultimately, after considering different research approaches, the CAB selected a randomized controlled trial (RCT) design as the most rigorous evaluation approach to support future sustainability and scaling. The Family Spirit RCT conducted between 2006 and 2011 found that the paraprofessional-delivered intervention promoted effective parenting, reduced maternal risks, and improved child developmental outcomes [21]. The long-term impact of Family Spirit with families is now being evaluated through a long-term follow-up (LTFU) study to be conducted between 2023 and 2028.

Critical knowledge gaps remain in understanding the long-term effectiveness of early childhood home-visiting programs. There are only two LTFU (15 + years) studies of home visiting RCTs in the US, neither conducted with NA communities [22, 23]. Additionally, no LTFU has assessed substance use or suicide impacts directly, and none has shown long-term efficacy of paraprofessional home visitors—a critical workforce for under resourced communities. Both substance use and suicide are leading causes of mortality for NA communities and comprise the largest public health disparities compared to all other racial and ethnic groups nationally [24]. As such, the Family Spirit Program now has an unprecedented opportunity to assess whether a culturally tailored early childhood home-visiting intervention provides long-term protection during a timeframe of peak mortality risk.

### Study objective and aims

This 16-year LTFU of the Family Spirit intervention RCT (NIDA R01 DA019042-01A1; ClinicalTrials.gov: NCT00373750) aims to determine if Family Spirit’s early impacts resulted in long-term protection against problematic substance use and suicide during peak risk periods. The study is part of a larger Center of Excellence grant funded by the National Institute on Drug Abuse, which aims to understand cultural, strengths-based approaches to substance use. Study aims include:

- Aim 1: Identify the long-term impact of Family Spirit on mothers’ substance use and suicidal thoughts and behaviors.
- Aim 2: Identify long-term impact of Family Spirit on adult children of the original participating mothers’ substance use and suicidal thoughts and behaviors.
- Aim 3: Explore the experience and long-term impacts of Family Spirit, specifically individual and intergenerational substance use, suicidal thoughts and behaviors, and related consequences through qualitative data collection from a purposive sample of children and mothers in both arms of the original trial.

## Methods

### Study design

This study is a LTFU of an RCT investigating the impact of Family Spirit for mothers and their now adult children in the communities that took part in the original trial. This study will use an explanatory mixed methods approach. We will use quantitative surveys and electronic health record (EHR) reviews to understand the Family Spirit intervention’s long-term impacts on maternal and child substance use, suicide, and related consequences, as well as mediators (i.e., maternal mental health, substance use, parenting behaviors) and moderators (i.e., mothers’ and children’s history of cultural loss; discrimination; and/or positive cultural identity and engagement) of these outcomes. Semi-structured, in-depth, individual interviews with a purposively sampled group of participants will be used to understand the Family Spirit intervention’s long-term impacts on individual and intergenerational experiences surrounding substance use, suicide, and related consequences.

### Study settings

The original and current study settings include four rural, reservation-based communities in Arizona that represent three distinct Tribal nations: the White Mountain Apache Tribe, San Carlos Apache Tribe and Navajo Nation. All three of these Tribes have taken part in collaborative research and service projects with the Johns Hopkins Center for Indigenous Health (formerly Center for American Indian Health) since the 1980s, and have been directly involved in the development, evaluation, and refinement of the Family Spirit Program since the mid-1990s. These settings share similar histories of historical trauma brought on by colonization, high rates of poverty, teen pregnancy and early exposure to substance misuse. Our community/tribally-based participatory approach to partnership with each of these communities will be leveraged through each phase of the study to ensure its success, from recruitment and enrollment through to dissemination of findings back to community members.

### Original trial participants

The original Family Spirit intervention RCT, conducted between 2006 and 2011, recruited *n* = 322 expectant NA mothers during pregnancy. They were 14–19 years old at the time of conception and recruited by 32 weeks’ gestation. The enrolled sample size by study site was n *=* 105 in the White Mountain Apache community, *n* = 83 in Fort Defiance (Navajo Nation), *n* = 69 in Tuba City (Navajo Nation), and *n* = 65 in the San Carlos Apache community. At time of recruitment for the LTFU study, mothers will range in age from 30 to 39, and their children will be 18–19 years old, respectively.

#### Eligibility criteria for LTFU study

To be included, individuals must have been enrolled in the original Family Spirit RCT and currently be available to participate in assessments in-person or virtually (for those who have moved away from the study community and cannot be reached in person). Participants will be excluded if they are experiencing challenges that preclude full participation in the consent or assessment protocol. For mothers or children who are deceased, we have obtained Tribal, Indian Health Service (IHS), and Johns Hopkins University Institutional Review Board (IRB) approvals to seek vital records to document causes of death. To obtain these data, we will partner with the Centers for Disease Control and Preventions’ National Death Index (NDI), which securely connects researchers to vital records of study participants.

#### Randomization

The original randomization status was 1:1 randomization to Family Spirit + Optimized Standard Care (Intervention) or Optimized Standard Care (OSC) alone (Control). Participants were stratified according to site, age (dichotomized at 12–15 and 16–19 years) and history of previous live births (dichotomized to 0 and ≥ 1) and then randomized to balance numbers in each group [25].

### Family Spirit logic model and theory of change for the LTFU study

Family Spirit’s logic model, published in 2012 [20], depicted how the intervention was designed to have immediate impacts on positive parenting and maternal behavioral and mental health risks that would otherwise disrupt positive parenting. Based on a well-accepted developmental theory put forth by Patterson (1989), the model posits parenting as the critical link between parents’ personal characteristics and environmental context and their children’s developmental outcomes [26].

**Fig. 1:**
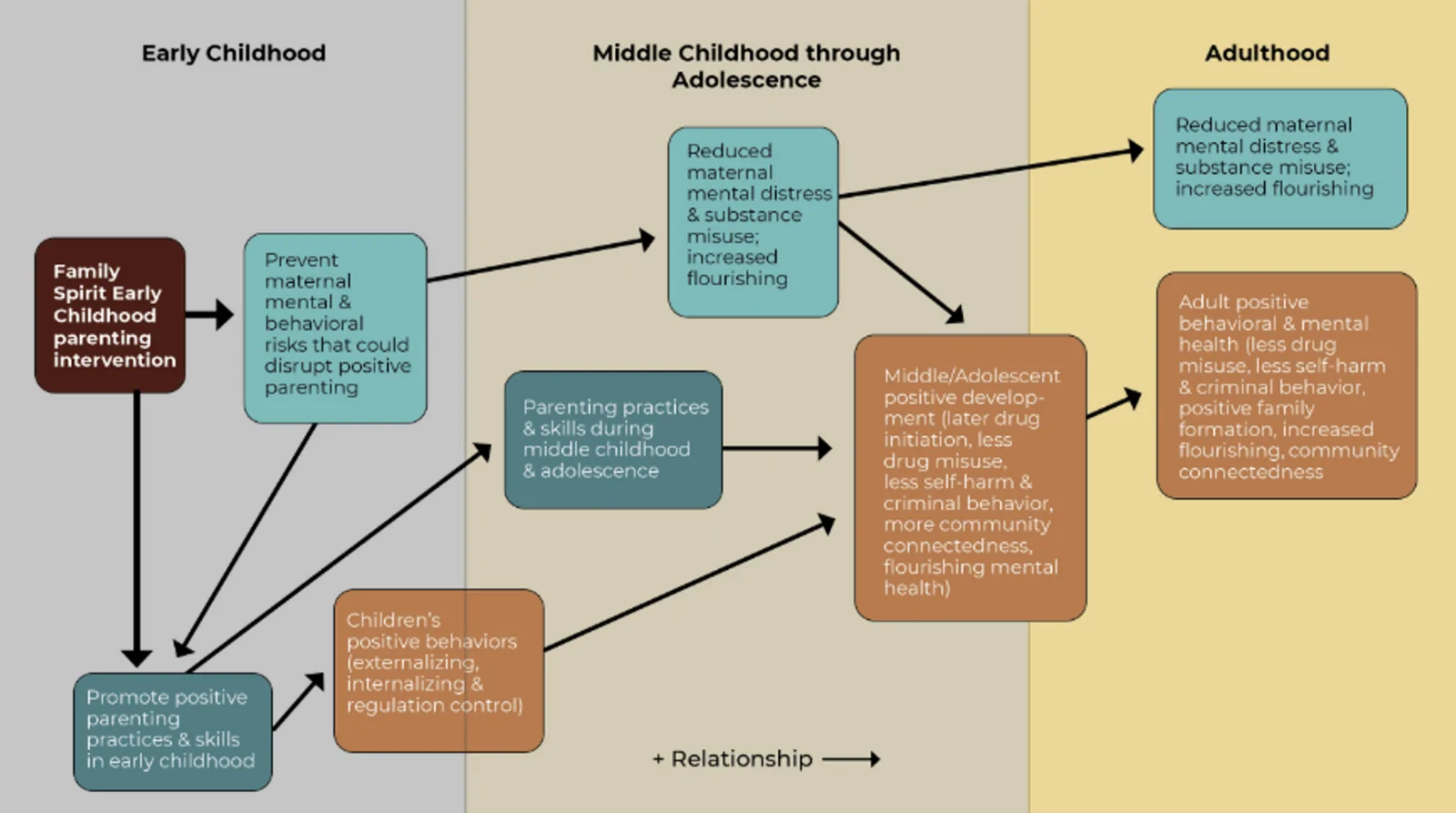
Theoretic developmental pathways of family spirit on long-term outcomes

A large body of research has demonstrated that ineffective parenting practices in early childhood leads to behavioral and mental health problems in middle and later childhood, including maladaptive peer group formation, conduct problems, drug use, sexual risk-taking, depression, anxiety, and suicidal risk in adolescence [27, 28]. Family Spirit’s aim to promote positive parenting in the short-term, while addressing maternal risks (substance use and depression) that could interrupt positive parenting, was intended to reduce infants’ and toddlers’ internalizing, externalizing, and regulatory problems, and promote positive behavioral and mental health outcomes through their middle and later childhood. Specifically, as depicted in Fig. 1, Family Spirit’s early intervention is expected to produce long-term impacts on prevention of problematic substance use and suicide for both mothers and children, while promoting positive family function, increased flourishing and mental health [20].

### Original trial: intervention condition – Family Spirit

The Family Spirit intervention in the original trial included 43 structured lessons delivered between pregnancy – 3 years postpartum. The intervention included a home visit structure loosely based on “Healthy Families America,” which was selected by community advisors for its cultural relevance. Healthy Families America consists of 12 key home visiting principles regarding client service and case management. It does not include specific intervention content [29]. Key curricular content for Family Spirit was derived from: (1) community input that guided content elements and cultural relevance; (2) childcare and parenting information from the *American Academy of Pediatrics’ Caring for Your Baby and Child: Birth to Age 5* [30], and (3) a review of successful home visiting practices. Family Spirit content was pilot tested through two studies between 2002 and 2005 and found to be a promising approach for improving maternal knowledge and involvement, as well as infant behavior outcomes [31–33]. In addition, during the 2006–2011 trial period, we received an administrative supplement (NIDA 3R01DA019042-03S1) to add curriculum and assessment strategies to address high rates of methamphetamine and other drug use in our study population.

### Original trial: control condition – Optimized Standard Care (OSC)

OSC, the control condition for the original trial, included assisted transportation to seven prenatal visits and nine well-baby visits for mothers during the first two years of their child’s life. When children turned two years of age, and well-baby visits diminished to one-year intervals, we added quarterly Family Assistance Visits and Referrals, in which home visitors conducted a “family service needs” questionnaire and then connected mother and family members to appropriate community services. Both intervention and control groups received the control condition, so we could examine the added benefits of Family Spirit over and above OSC.

### Recruitment and consent for LTFU study

We will attempt to locate and re-consent all mothers and children who consented to participate in the original trial. For this study, we obtained a Health Insurance Portability and Accountability Act (HIPAA) waiver to enable verification of contact information in the electronic health records (EHR) at hospitals in the participating communities, based on medical record numbers and dates of birth for mother and child participants in the original trial that were retained for long-term follow-up purposes, with ethics board approval.

Once contact information is confirmed, local research staff will attempt to reach potential adult participants via home visit, phone/text, email, or approved social media sites. Research staff will then describe study aims and activities, and if participants are interested, they will conduct informed consent for both self-report questionnaires and chart reviews (Aims 1 and 2). Participants may indicate in the consent if they are willing to be contacted later in the study to complete a qualitative interview, should they be purposively selected for that component (Aim 3). A separate consent form will be used for the qualitative interview. Mothers will also be asked for permission to contact their adult child who participated in the original trial. If mothers agree, study staff will ask for assistance in locating their child and attempt to conduct consent with the child once they turn 18 years old. If a mother does not give permission to contact their child, the adult child will not be contacted. If the mother is deceased, the adult child will be approached independently to consent voluntarily, once they are 18 years old.

### Ethics approvals

We have obtained all required community-, regional-, and national-level tribal health board, tribal council, IHS, and Johns Hopkins University IRB approvals necessary for this research with the Navajo Nation, White Mountain Apache Tribe, and San Carlos Apache Tribe. Participants’ privacy is protected by a Certificate of Confidentiality.

### Role of the funding source

The study sponsors had no role in study design, data collection, data analysis, data interpretation, or writing of the report.

### Primary outcomes

Our primary outcomes include rates of lifetime substance use, medical discharges related to substance use and suicidal thoughts and/or behaviors (as measured by diagnostic codes), comparing mothers randomized to Family Spirit vs. OSC. For adult children, primary outcomes include time (in years) to initiation of substances, and past year substance use rates comparing children whose mothers were randomized to Family Spirit vs. OSC. We will also explore secondary outcomes, including maternal and child overdose and suicide death rates. Data for primary and secondary outcomes will come from self-report questionnaires, EHR reviews, and the National Death Index.

### Data collection and measures

#### Quantitative assessments (Aims 1 and 2) (Table 1)

Cross-sectional, self-report questionnaires will be used. Measures were selected based on psychometric properties and cultural appropriateness in the participating communities and have been reviewed line-by-line prior to use by local team members. After completing consent, data collectors will schedule an assessment visit in a private setting that is most convenient for the study participant (i.e., study office, participant’s home, or study vehicle). Participants will complete assessments on a tablet using REDCap [34]. Data collectors will remain present to assist with any challenges.

**Table 1:** Measures for Aim 1 and Aim 2, organized by outcome

Domain/Measure	Number of Items	Mothers (M) or Adult Children (AC)	Estimated Completion Time
Demographics	31 (M) 26 (AC)	M & AC	10–15
*Primary Outcomes*			
Substance Use History [35]		M & AC	
Suicide History [36, 37]	10	M & AC	2
Lifetime Depression Assessment – Self-Report (LIDAS) [38] Lifetime Anxiety Questionnaire	35 16	M & AC M & AC	10 10
Medical Events involving Substance Use/Suicide: EHR Reviews	N/A	M & AC	N/A
*Secondary Outcomes*			
Strengths and Difficulties Questionnaire (SDQ) [39]	25	M & AC	15
Criminal Justice Involvement [35]	20	M & AC	5
Holistic Health [35]	4	M & AC	1
Flourishing – Diener Flourishing Scale [40]	8	M & AC	2
*Mediators*			
POSIT – Problem Oriented Screening for Teenagers [41]	11	M & AC	2
Perceived Stress Index [42]	4	M & AC	1
Suicide History (Lifetime, 1 Year, 1 month) [36, 37]	*Noted above		
*Moderators*			
Lifetime Discrimination Scale [43]	14	M & AC	2
Benevolent and Adverse Childhood Experiences (BCE/ACEs) [44, 45]	30	AC	7
Cultural Identity & Engagement in Cultural Routines [46]	24	M & AC	5
Intergenerational Connectedness [47]	5	M & AC	1
Depression (CES-D) [48]	20	M & AC	5
Anxiety – GAD-7 [49]	7	M & AC	1

#### EHR reviews

Participants’ consent to access medical records will be obtained through a medical record release form. For this study, we have also confirmed that: (1) our participants’ data from previous paper charts have been uploaded into EHRs, or (2) there is access to previous paper charts as needed. Participants’ EHRs will be reviewed by study staff members with expertise in chart extraction. A templated data extraction form will be used to document information on dates and IDC-10 codes for any clinical or emergency department visits, discharges, hospital admissions, and/or diagnoses related to substance use or suicidal thoughts/behaviors. For mothers, EHR reviews will span from the date of enrollment in the original trial until age 31 (the lowest possible age for LTFU participants) to ensure consistency of findings. For adult children, EHR reviews will span from birth to age 19, an age all eligible children will reach during the study period.

#### Qualitative interviews (Aim 3)

Participants who are purposively sampled and provide consent will be asked to complete an in-depth interview in-person or via phone/video chat (for participants who live outside of the participating communities). The interview guide will focus on Family Spirit intervention’s long-term impacts on individual and intergenerational experiences surrounding substance use, suicide, and related consequences. Interview guides may also elicit experiences surrounding maternal-child relationships, cultural connection, and protective factors that mitigate or reduce adverse health outcomes.

### Sample size calculations

#### Quantitative assessments (Aims 1 and 2)

The original Family Spirit trial was powered to detect a clinically meaningful effect size (ES ≥ 0.3) for primary outcomes [20]. Approximately 17% of participants dropped out by the child’s 3rd birthday. We thus based our sample size calculations on a conservative 25% attrition rate from the baseline enrollment at the LTFU period, yielding a proposed *n* = 242 mothers and *n* = 242 children across the two experimental conditions. With power set to 80% and α = 0.05, we expect to be able to detect a difference in rate ratio of the outcome (e.g., rate of suicide emergency department-related visits) between the two groups of 36% or higher (small to medium effect size) and a hazard ratio of approximately 0.69, or a medium to low relative difference for the outcome looking at adult children’s age of initiation of substance use.

#### Qualitative interviews (Aim 3)

We will purposively sample participants based on how they self-report substance use, as measured through Aim 1 and Aim 2 analyses, in order to represent a continuum of substance use behaviors ranging from abstinence through problematic use. We will also attempt to balance recruitment of participants for qualitative interviews by sites, type of participant (mothers and adult children, including an even number of women and men) and experimental condition in the original trial (Family Spirit vs. Optimized Standard Care). We anticipate a sample of up to *N* = 96 (48 mothers + 48 adult children). Given our stratified recruitment approach, data saturation will be monitored throughout data analysis.

### Data analysis

#### Quantitative analysis (Aims 1 and 2)

The four primary hypotheses we will test are:

1. Mothers who were randomized to the Family Spirit intervention will have lower rates of lifetime substance use related medical discharges than mothers who were randomized to OSC (Aim 1).
2. Mothers who were randomized to the Family Spirit intervention will have lower rates of lifetime suicidal thoughts and/or behaviors than mothers who were randomized to OSC (Aim 1).
3. Children whose mothers were randomized to the Family Spirit intervention will initiate use of substances later than children whose mothers who were randomized to OSC (Aim 2).
4. Children whose mothers were randomized to the Family Spirit intervention will have lower past year incidence of substance use than children whose mothers were randomized to OSC (Aim 2).

Secondary hypotheses include understanding Family Spirit benefits to mothers’ and children’s emotional, social, and behavioral functionality; children’s history of adverse and benevolent childhood experiences; children’s placement in foster care; children’s/mothers’ criminal justice histories; and children’s/mothers’ relational health.

For Aims 1 and 2, initial analyses will be conducted under an “intent-to-treat” model, in which data are analyzed according to assignment at randomization [50, 51]. Our study consists of self-report and EHR data measured longitudinally; thus, our analytical approaches account for potential biases related to loss to follow-up, treatment switching, and truncation by death [52]. The underlying structure of outcome variables for our primary hypotheses also varies; hypotheses 1, 2, and 4 involve count-based variables and require Poisson regression approaches that adjust for varying follow-up time, while hypothesis 3 involves a time-to-event model that requires a survival analytic approach.

#### Model building & estimation for primary outcomes

For hypotheses 1, 2, and 4, outcomes will be tallied by counting the number of medical discharges and/or days (self-report data) using substances, where each outcome is documented over time for each participant. These counts will be graphed to inspect overdispersion and excessive number of zeros to ensure assumptions of a Poisson model are met. Count data will then be merged with baseline Family Spirit data. Initial outcome models will be specified and include the outcome variable regressed on the experimental condition indicator (i.e., 0 = OSC; 1 = Family Spirit). We will then run models that control for varying amounts of exposure time/study site and then add adjustments for differences in baseline characteristics between groups. Our primary outcome for these three hypotheses will be the difference in incidence rate ratios between Family Spirit and OSC in the unadjusted analysis. For hypothesis 3, we will use survival analysis to measure the difference in self-reported age of initiation of substances between children exposed to Family Spirit vs. OSC. Since all children in the trial were enrolled until 36 months of age, time will be measured starting at 3.5 years of age and in 3-month age intervals. We will graphically explore the hazard rate across participants and then view Kaplan-Meier curves stratified by experimental condition to examine average time (in age) to initiation of substances. To examine statistically significant (i.e., *p <* 0.05) differences in time to initiation of substances between conditions, Cox proportional hazard models will be fit. Before fitting the models, we will explore whether the proportionality assumption is met by examining Schoenfeld residuals and adjust models accordingly (through stratification) to account for non-proportionality. The primary outcome will be the relative risk (or hazard ratio) of earlier initiation between conditions. Like our Poisson models, additional covariates (exposure time, site, and baseline characteristics) will be added in a secondary adjusted model.

Secondary outcome analyses will focus on similar models using regression or survival analyses as appropriate. For self-report data among mothers, the proposal incorporates longitudinal measurement, which leads to a nested data structure. The data will have a 2-level structure, with nine repeated measurements (level 1) nested within participants (level 2). As such, we will utilize mixed-effects regression models [53]. These models are highly flexible, addressing variability in frequency and spacing of measurements, outcome distributions (continuous; counts), and linear and non-linear patterns of change.

#### Model building & estimation for secondary outcomes

Secondary outcomes will be graphically and descriptively evaluated to determine the best distribution for modeling using “spaghetti plots.” [54] Singer and Willett’s model building approach [55] will be used: The first model estimates the proportion of outcome variance at each level, and the second model adds growth terms. The third model adds fixed-effect indicators to test for differences in baseline characteristics. If applicable, differences will be controlled for in the models that follow and include site. Sites are modeled as fixed effects to overcome potential systematic variation and confounding at the site level. The fourth model adds the experimental condition indicator (e.g., 0 = OSC; 1 = Family Spirit) and cross-level interaction effects between condition and level 1 (time) terms. We will measure between-group differences in each outcome specified through an interaction term.

#### Exploration of mediators and moderators

Our selection of mediators and moderators is again guided by our theory of change. Due to limited sample size, mediator and moderator analyses will be exploratory. Proposed mediators include parenting practices and behaviors. Proposed moderators include basic demographics. For mediation, we will calculate the change between baseline and the original trial end date and include this as a main effect in the model; a Sobel-Goodman test [51] will be run to determine the degree to which changes in parenting practices mediated the effect of Family Spirit on mothers’ and children’s later substance use and suicidal thoughts and behaviors (primary outcome for mothers; and secondary outcome for adult children). For moderation analyses, we will use a joint effects approach by including a three-way interaction between Family Spirit/control, time point, and the moderator of interest. A significant interaction suggests that the effects of the intervention vary according to the presence of the moderator of interest. We calculated power for a multivariate linear regression analysis with a sample size of *N =* 242 (based on stratified analyses by participant type). In a model with two independent variables (Family Spirit and mediator variable) we would have sufficient power (0.80) to detect a small-to-moderate effect size for mediating independent variables and interaction effects (Cohen’s f^2^ = 0.05).

#### Qualitative analysis (Aim 3)

Interviews will be audio-recorded and then transcribed for data analysis. Coding will be employed through a hybrid approach that uses both inductive and deductive coding strategies. Hybrid coding involves the creation of theory- and data-driven codes and sub-codes generated from deductive and inductive coding processes [56]. Deductive coding is a top-down method in which codes are determined by a particular theoretical framework [57] – i.e., codes will be developed a priori based on the research questions posed. Inductive coding is a bottom-up approach where codes are identified through textual data searching for domains and themes to make meaning; therefore, codes are developed *posteriori* [57, 58]. A codebook will be developed to include both primary codes (encompass main idea(s)) and secondary codes (subcodes that branch off primary codes). The codebook will be a living document to track the iterative coding process and ensure validity through the outlining and verification of primary and secondary codes [59].

The inclusion of multiple coders will ensure reliability [60, 61]. Dedoose, a qualitative data analysis software, will be used for data management and analytic procedures. Hybrid coding is an iterative process, as, continued conferencing, code naming, definitions, and application will shift throughout analysis through conferencing among coders [59, 62].

A thematic analysis approach will be employed, which involves developing, analyzing and interpreting patterns across a qualitative dataset [63]. Reflexive thematic analysis located within a qualitative paradigm emphasizes the active role of the study team in coding and theme development, acknowledging a subjective process. This analysis approach comprehensively highlights stories and experiences as narratively expressed through the voice of participants, while acknowledging the positionalities of researchers as interpreters and translators of the data.

The research team will thoroughly review the hybrid coded transcripts independently and then discuss themes that emerge across all transcripts, following the six guiding steps for reflexive thematic analysis [62]. Aggregated and synthesized data will be shared and reviewed by study staff, who are members of and reside within partnering communities. Their direct experiences and relationships with participants will provide verification and validity of qualitative findings and inform culturally aligned data dissemination.

#### Mixed methods integration

Our mixed-methods analysis will focus on understanding heterogeneous effects of Family Spirit and mediating pathways toward different trajectories. Following an explanatory mixed methods approach, we will purposively sample subgroups based on substance use histories reported, and then further explore their histories through qualitative in-depth interviews. The data will then be mixed to holistically compare subgroups through an integrated joint display [64].

#### Attrition and missing data

Key threats to the LTFU study include non-adherence/non-compliance, the potential that OSC participants obtained Family Spirit after the trial (i.e., “treatment switching”), loss to follow-up, and truncation by death or other events. Based on our knowledge of Family Spirit dissemination locations, we expect minimal treatment switching, but we will track this as part of a sensitivity analysis. For loss to follow-up, based on the amount of missing data and evidence of missingness related to randomness, we will address missingness through ML-based estimation or multilevel multiple imputation [65]. Truncation by death is a particularly unique consideration in the proposed study given our focus on non-mortality outcomes; we will therefore estimate the survivor average causal effect [66].

## Discussion

This paper outlines the protocol for a 16-year LTFU of the Family Spirit home-visiting intervention trial, with mothers now aged 30–39 years old (the highest risk period for premature mortality linked to substance misuse) and their children now 18–19 years old (an age group when substance use patterns have been established and suicide risk is highest in NA communities) [65, 67]. NA women of childbearing age have the highest overdose rates of any female racial group in the US – now twice the rate of White women ages 25-44 [68]. Recent stressors and treatment access issues exacerbated by the COVID-19 pandemic further widened Indigenous inequities in drug overdose deaths, including for mothers [69].

The original Family Spirit RCT demonstrated that mothers who received the intervention had significantly less drug use and depressive symptoms from birth to three years postpartum. Their children had significantly lower early externalizing and internalizing problems than children in the control group [18]. Since the original trial, the study team has established clear links between substance use and suicide thoughts/behaviors among youth in the participating communities, including high rates of co-occurrence and shared risk and protective factors [4, 70, 71]. The intersection between substance use and suicide has been observed among many other US subgroups, with national advocates calling for cohesive prevention approaches to address both [72]. Suicide that concentrates in youth or during active parenthood years translates to high burdens of productive years of life lost and devastating, intergenerational despair – thus, becoming a catalyst for next generation risk.

### Limitations

The main limitation is the possibility that we will not enroll an adequate and representative sample to measure the proposed outcomes. The study team has a successful history with LTFU and proven competency to achieve study recruitment goals [73, 74]. In addition, the original trial design included key factors that paved the way for LTFU, including persistent community trust relationships, sustained employment and professional growth of NA team members who worked on the original trial, and a logic model devised for the original study that hypothesized long-term impacts on substance use and mental health trajectories. Our study population predominantly uses their local hospital for inpatient and outpatient care, which will aid in collecting thorough EHR data for primary outcomes.

A secondary limitation is the temporality of the measures. Measuring age of initiation of drug use for children at age 18 may be affected by measurement bias when the onset of drug use occurred long before the measurement. However, previous research confirms that self-report of substance use is valid and more accurate than lab tests or reports from family members [11]. As with any self-reported data collection, there is a potential for recall bias. Participants may recall events or behaviors from years prior with varying levels of accuracy. To mitigate these limitations, data extraction from EHRs, combined with quantitative and qualitative self-reports across two generations, should create a comprehensive picture of participants’ substance use and suicide histories, risk and protective factors, and relative benefits from Family Spirit.

## Conclusion

Our study holds promise to demonstrate how a culturally tailored, early childhood intervention may interrupt devastating mortality trends by building upon Indigenous strengths, providing a model for promoting well-being across the life course. The ages of the mothers and adult children at the time of LTFU will allow us to understand Family Spirit’s impacts on both drug use and suicide during peak risk periods in communities that have endured high rates of suicide behaviors for more than 40 years. Given Family Spirit’s past success and proven scalability, determining its long-term potential to close gaps in substance use and suicidal thoughts/behavior for NA parents and youth has strong implications for all NA families and communities.

## Data Availability

To protect the privacy of study participants, a Certificate of Confidentiality through the National Institutes of Health (NIH) for the study has been obtained. Due to the Certificate, investigators and others who have access to research records will not disclose identifying information except when the participant consents or in certain instances when federal, state, or local law or regulation requires disclosure. NIH expects investigators to inform research participants of the protections and the limits to protections provided by a Certificate issued by this Policy.

## Abbreviations

NA: Native American
LTFU: Long-term Follow-up
RCT: Randomized Controlled Trial
IRB: Institutional Review Board
HER: Electronic Health Record
